# Automated Intracranial Clot Detection: A Promising Tool for Vascular Occlusion Detection in Non-Enhanced CT

**DOI:** 10.3390/diagnostics13182863

**Published:** 2023-09-05

**Authors:** Ricarda Schwarz, Georg Bier, Vera Wilke, Carlo Wilke, Oliver Taubmann, Hendrik Ditt, Johann-Martin Hempel, Ulrike Ernemann, Marius Horger, Georg Gohla

**Affiliations:** 1 Department of Diagnostic and Interventional Radiology, Eberhard Karls University of Tuebingen, D-72076 Tuebingen, Germany; ricarda.schwarz@med.uni-tuebingen.de (R.S.); marius.horger@med.uni-tuebingen.de (M.H.); 2 Department of Diagnostic and Interventional Neuroradiology, Eberhard Karls University of Tuebingen, D-72076 Tuebingen, Germany; georg.bier@uni-tuebingen.de (G.B.); johann-martin.hempel@uni-tuebingen.de (J.-M.H.); ulrike.ernemann@med.uni-tuebingen.de (U.E.); 3 Radiologie Salzstraße, D-48143 Muenster, Germany; 4 Department of Neurology & Stroke, Eberhard Karls University of Tuebingen, D-72076 Tuebingen, Germany; vera.wilke@med.uni-tuebingen.de; 5 Centre for Neurovascular Diseases Tübingen, D-72076 Tuebingen, Germany; 6 Division Translational Genomics of Neurodegenerative Diseases, Hertie Institute for Clinical Brain Research, Center of Neurology, University of Tuebingen, D-72076 Tuebingen, Germany; carlo.wilke@med.uni-tuebingen.de; 7 German Center for Neurodegenerative Diseases (DZNE), D-72076 Tuebingen, Germany; 8 Siemens Healthcare GmbH, Computed Tomography, D-91301 Forchheim, Germany; oliver.taubmann@siemens-healthineers.com (O.T.); hendrik.ditt@siemens-healthineers.com (H.D.)

**Keywords:** deep learning, non-enhanced brain CT, intracranial arterial vessel occlusion

## Abstract

(1) Background: to test the diagnostic performance of a fully convolutional neural network-based software prototype for clot detection in intracranial arteries using non-enhanced computed tomography (NECT) imaging data. (2) Methods: we retrospectively identified 85 patients with stroke imaging and one intracranial vessel occlusion. An automated clot detection prototype computed clot location, clot length, and clot volume in NECT scans. Clot detection rates were compared to the visual assessment of the hyperdense artery sign by two neuroradiologists. CT angiography (CTA) was used as the ground truth. Additionally, NIHSS, ASPECTS, type of therapy, and TOAST were registered to assess the relationship between clinical parameters, image results, and chosen therapy. (3) Results: the overall detection rate of the software was 66%, while the human readers had lower rates of 46% and 24%, respectively. Clot detection rates of the automated software were best in the proximal middle cerebral artery (MCA) and the intracranial carotid artery (ICA) with 88–92% followed by the more distal MCA and basilar artery with 67–69%. There was a high correlation between greater clot length and interventional thrombectomy and between smaller clot length and rather conservative treatment. (4) Conclusions: the automated clot detection prototype has the potential to detect intracranial arterial thromboembolism in NECT images, particularly in the ICA and MCA. Thus, it could support radiologists in emergency settings to speed up the diagnosis of acute ischemic stroke, especially in settings where CTA is not available.

## 1. Introduction

Worldwide, ischemic stroke is the second leading cause of death; thus, representing a significant health problem [1]. Morbidity and mortality in stroke patients highly depend on early and effective therapy. Non-enhanced computed tomography (NECT) is an integral part of the standardized stroke imaging protocol. It is primarily used to exclude hemorrhage, but also for the identification of subtle, early, direct, and indirect findings suggesting embolism. The most important direct sign is the hyperdense artery sign (HAS), whereas subtle parenchymal hypoattenuation, loss of insular ribbon, cortical sulcal effacement, and obscuration of grey-white matter differentiation in the basal ganglia are indirect signs [2].

HAS is difficult to detect but has a high impact on the diagnosis, therapy, and prognosis of stroke. It is the first correlate of ischemic stroke and is highly specific for complete vessel occlusion as the high attenuation values in the vessel are indicative of an acute thrombus [3,4,5]. NECT can provide a direct correlate for the clot. In contrast, computed tomography angiography (CTA), CT perfusion, or interventional angiography can only show the vessel occlusion as an indirect correlate for a clot, which cannot supply further clot characteristics. Clot characteristics, e.g., Hounsfield unit (HU) values, clot composition, clot volume, clot burden, and exact clot location may give important additional information on etiology, prognosis, and therapeutic success [6,7]. Lower clot burden, smaller thrombus length, and more distal thrombus location are predictors of a better clinical and radiologic outcome [8,9,10]. Hyperdense clots are erythrocyte-rich and respond better to mechanical thrombectomy [11,12,13]. In contrast, isodense clots are rich in fibrin with increased elasticity and stiffness [14,15] and are associated with unsuccessful reperfusion by mechanical thrombectomy [16]. These isodense fibrin-rich clots are more responsive to pharmacologic thrombolysis [17]. Therefore, clot characteristics may alter the therapeutic strategy for large vessel occlusion, suggesting that a more comprehensive endovascular approach, including the use of intra-arterial pharmacological thrombolytics, should be considered in the presence of fibrin-rich isodense clots. However, the sensitivity of HAS is low, the characterization of a clot is even more challenging, and the absence of HAS cannot exclude vessel occlusion [3,10,18,19].

In emergency settings, such as acute stroke, prompt decision-making is paramount. A delay in treatment initiation can have a significant impact on the patient’s prognosis. Nonetheless, the identification of a thrombus within smaller cerebral vessels or in individuals exhibiting pre-existing neurological alterations necessitates a significant amount of time, even for experienced radiologists or neuroradiologists. An automated CT scan assessment tool could assist radiologists in clot detection and assess relevant clot characteristics. Research about automated clot detection in NECT of acute ischemic stroke is rare and in most of the published studies is not compared to clinical data [20,21,22].

Hence, the purpose of our study was to test the diagnostic performance of a prototype tool for automated clot detection and characterization in NECT scans and relate the results to clinical parameters and chosen therapy.

## 2. Materials and Methods

### 2.1. Study Design

**Figure 1:**
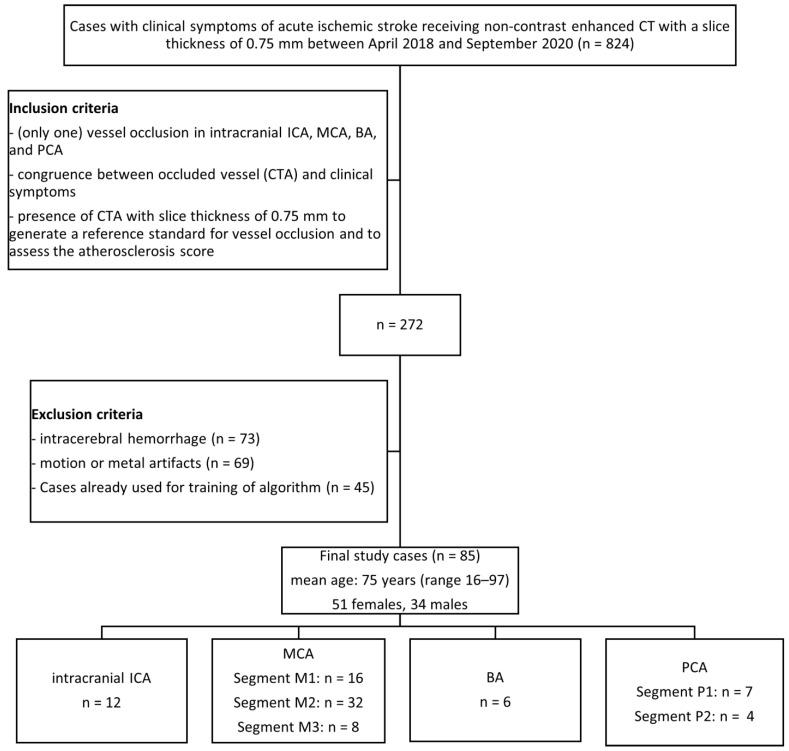
Flow chart with patient selection criteria. ICA = internal carotid artery; MCA = middle cerebral artery; BA = basilar artery; PCA = posterior cerebral artery; CTA = CT angiography.

The local institutional ethics committee approved this retrospective data evaluation (587/2019BO2). We performed a retrospective database search between April 2018 and September 2020. This study was conducted according to the STARD guidelines (Standards for Reporting of Diagnostic Accuracy Studies) [23]. The inclusion and exclusion criteria are detailed in Figure 1.

### 2.2. CT Image Analysis

One neuroradiologist (M.H., reader 1) with 30 years and one radiologist (R.S., reader 2) with 5 years of experience in cerebral imaging, interpreted the randomized non-enhanced 0.75 mm thin-slice image of the axial brain CT of the included patients regarding the presence and location (side and segment) of HAS. The intracranial internal carotid artery (ICA) occlusion was detected beyond the passing the carotid channel in the temporal bone; the middle cerebral artery (MCA) segments (MCA1 up to the upward point of the Sylvian fissure, MCA2 up to the top of the Sylvian fissure, MCA3 up to the cortical surface); the posterior cerebral artery (PCA) segments PCA1 and PCA2; and the basilar artery (BA). The readers were blinded to clinical history and imaging reports. This evaluation was repeated three months later to evaluate the intra-observer agreement. In a different session with a time difference of more than six months, reader 1 evaluated the corresponding CTA of the same patients to generate reference standard interpretations for vessel occlusion. In this reading, for statistical correlation, the degree of calcification in intracranial arteries was also assessed using the atherosclerosis score published by Chen 2006 and Woodcock 1999 [24,25].

### 2.3. Automated Clot Detection Tool

The software is a prototype and not a commercially available product (Siemens Healthineers, Forchheim, Germany); only non-contrasting data was used to find the clot. The technique of automatic thrombus detection relies on a convolutional neural network trained on an independent multicenter data set consisting of thin-slice NECT scans of 664 patients suffering from acute ischemic stroke. Clots were circled in these test data sets so that the software could learn what a clot is. In an internal cross-validation performed on the training data, the clot was found in 616 (92.8%) of 664 patients among the five predicted candidates with the highest clot candidate scores when the affected hemisphere was assumed to be known and in 603 (90.8%) when it was not.

**Figure 2:**
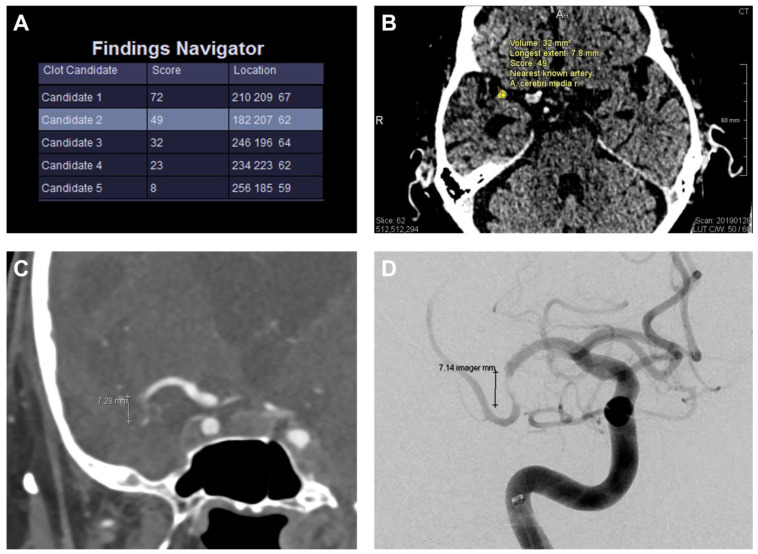
Clot detection prototype with clot evaluation. The tool shows the most likely clot candidates (with a maximum of 5) ordered by the clot candidate score (“Score”, scale range 0–100), and the corresponding location in CT using a coordinate system (**A**). The clot candidate score is determined by the maximum heat map value (“per-pixel likelihood”). Higher values indicate a stronger resemblance to a true clot sign. The different candidates can be manually selected to be displayed in the CT images. In this case, “Candidate 2” represents the true clot and is illustrated in (**B**). Random walk segmentation, which is initialized automatically based on the heat map, is run to obtain the clot contour for each candidate. The clot is contoured in the displayed slice (yellow dots, **B**), and the estimated volume and longest extent (7.8 mm) are displayed next to it. (**C**) shows the correlating CT angiography and (**D**) the cerebral angiography of the same patient as in (**A**,**B**) with a nearly similar length of the occluded vessel segment (CT angiography: 7.28 mm; cerebral angiography: 7.14 mm vs. 7.8 mm in non-enhanced CT (**B**)).

The model input comprises several channels and the original image, such as a left/right difference image and a vessel probability map obtained from a brain atlas. The model output is a voxel-level heatmap indicating potential clots from which candidate positions are derived. According to the model, the clot detection prototype displays the most likely clot candidates (with a maximum of 5), as shown in Figure 2.

They are ordered by the correlating clot candidate score (CCS; scale range 0–100), which is determined by the maximum heat map value (“per-pixel likelihood”). Higher values indicate a stronger resemblance to a true clot sign. The neuroradiologist reviews the candidates and selects the correct true clot.

The random walk segmentation, which is initialized automatically based on the heat map, is run to obtain the clot contour for each candidate. This contour is, in turn, used to calculate the clot volume and estimate the length of the clot using principal component analysis.

### 2.4. Clinical Analysis

For all patients, the time between symptom onset and CT imaging was classified as <4.5 h, >4.5 h, or uncertain (wake-up stroke/symptom onset not observed). The National Institutes of Health Stroke Scale (NIHSS) was assessed immediately before the scan [26]. The clinically suspected stroke etiology was evaluated based on the patient’s history and technical investigations during the hospitalization using the TOAST criteria (Trial of Org 10,172 in Acute Stroke Treatment) [27]. The type of therapy chosen (exclusively supportive, intravenous thrombolysis, interventional thrombectomy/intraarterial lysis in combination or alone) was registered for each patient to assess the relationship between clinical parameters, image analysis results, and chosen therapy. In addition, the Alberta Stroke Early CT Score (ASPECTS) was calculated automatically.

### 2.5. Standard of Reference

The CTA was set as the ground truth for assessing vessel occlusion by the non-invasive imaging technique. However, clot length and volume measurements are not reliable in CTA. Therefore, the corresponding estimates issued by the prototype could not be correlated.

### 2.6. Statistical Analysis

Statistical analyses were computed using SPSS Version 27 (IBM Corp., Armonk, NY, USA). CTA was used as the ground truth for comparing the diagnostic accuracy of the human readers and the automated clot detection tool. A case was counted as true positive if there was congruence between the location of vessel occlusion in the CTA and the location of a positive HAS reported by the readers or one of the maximum five clot candidates suggested by the tool. Findings were considered false positives if readers reported a HAS at an incorrect location or if the true clot was not included among clot candidates suggested by the tool. The tool always suggests at least one clot candidate, resulting in no false-negative findings. There were no true-negative findings because we did not include patients without vascular occlusion. Therefore, we do not report true-negative and false-negative findings for the clot detection tool and the human readers. In addition, specificity and negative predictive value could not be calculated. We calculated the sensitivity and positive predictive value (PPV); *p*-values were determined using the chi-squared test.

The Shapiro–Wilk test was used to test for normal distribution. Spearman’s rank–order correlation coefficient was used to compare ordinal clinical parameters with imaging analysis. The phi coefficient investigated the correlation between nominal clinical parameters and imaging findings. Spearman’s correlation coefficient and phi coefficient r < 0.1 were interpreted as no correlation, 0.1 ≤ r < 0.3 as weak, 0.3 ≤ r < 0.7 as moderate, and r ≥ 0.7 as strong.

The significance level was set at 0.05 for all tests and displayed in the images as follows: * < 0.05, ** < 0.01; *** < 0.001. Inter-observer and intra-observer agreements were calculated for each imaging finding using Cohen’s kappa. A kappa of <0.4 was considered as poor, <0.75 as moderate, and ≥0.75 as an excellent agreement.

## 3. Results

### 3.1. Study Population

Eighty-five patients were enrolled in this retrospective study. The mean age of the patients was 75 ± 12 years (34 males, 51 females, range: 16–97 years). Based on the CTA assessment, the clot was located in the anterior circulation in 80% of the cases and the posterior circulation in 20%. Vessel occlusions of the left (*n* = 40) and right (*n* = 39) sides were represented with approximately equal frequency. The basilar artery was occluded in six cases. Detailed information on vessel distribution, time interval between symptom onset and imaging; and clinical analysis results including NIHSS, TOAST criteria, atherosclerosis score, and selected therapy are shown in Appendix A, Table A1.

### 3.2. Results of the NECT Analysis by the Readers

As shown in Table 1, true-positive and false-positive detection rates, sensitivity, and PPV of the HAS showed several differences between the two readers which lead to a poor inter-observer agreement of κ = 0.36.

Thus, the comparison of each reader’s first and second ratings revealed excellent intra-observer agreement (average κ = 0.93). Therefore, further statistical analysis was calculated with the first evaluation of each reader, but separately for readers 1 and 2.

### 3.3. Results of the Automated Clot Detection by the Prototype

True- and false-positive detection rates, sensitivity, and PPV of the clots suggested by the tool are displayed in Table 1. The accurate candidate was displayed in the first position in 33%, second in 15%, third in 8%, fourth in 5%, and fifth in 5% of cases. The correlating CCS, clot length, and volume as well as the automated ASPECTS are displayed in Table 2. For the distribution of true clots among clot candidates and corresponding CCS, see Appendix A, Table A2.

The displayed position of the clot candidate in the ranking issued by the tool revealed a highly significant negative correlation with the true-positive candidates (*p* < 0.001, r = −0.32); a clot candidate displayed in the first or second position was more often a true-positive finding. Moreover, the automatically calculated CCS revealed a highly significant positive correlation with the true-positive candidates (*p* < 0.001, r = 0.28).

### 3.4. Head-to-Head Comparison of the Tool with the Human Readers

**Figure 3:**
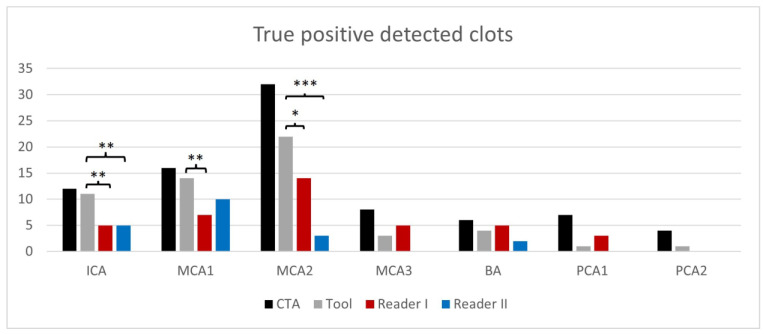
True-positive rates of clot detection according to occluded vessel and readers compared to the ground truth/reference standard computed tomography angiography (CTA). (* *p* < 0.05, ** *p* < 0.01; *** *p* < 0.001); intracranial internal carotid artery (ICA); proximal/middle/distal-middle cerebral artery (MCA1/2/3); basilar artery (BA); proximal/distal-posterior cerebral artery (PCA1/2).

Figure 3 displays the comparison of true-positive rates for clot detection between the readers, the tool, and the reference standard CTA.

The tool was significantly superior in detecting clots in comparison to the readers (*p* = 0.009 for reader one and *p* < 0.001 for reader 2). The occluded vessel was significantly superior in the detection of ICA/MCA1 and MCA2 clots (*p* < 0.01 in ICA for both readers and MCA1 for reader 1; *p* < 0.001 in MCA2 for reader 2 and *p* < 0.05 in MCA2 for reader 1). Like the readers, the tool correctly detected more clots in the anterior (readers 27–46%; tool 74%) than in the posterior circulation (readers 12–47%, tool 35%).

Unlike the readers, the tool output was not associated with the severity of the ischemic demarcation, as reflected by the ASPECTS. The smaller the ASPECTS (indicating a greater number of involved vascular territories/a higher volume of demarcation), the more likely it was that the readers correctly detected a clot (true-positive findings; reader 1, *p* = 0.012, r = 0.27; reader 2, *p* = 0.037, r = 0.23).

There was neither a significant correlation for the readers nor for the tool between the presence of a true-positive finding and the severity of atherosclerosis or the etiology of the clot (TOAST) or the clinical severity of the stroke (NIHSS) or the time point of imaging.

### 3.5. Clinical Use Case: Decision Support for ICA/MCA Recanalization

To simulate a realistic clinical emergency setting where diagnostic decisions are limited, we conducted an additional experiment in which only up to three clot candidate suggestions per patient were checked by the human reader. Moreover, the decision for an endovascular thrombectomy is more often made for clots in proximal arteries (ICA and MCA1) because of a better benefit–risk evaluation [28].

The automatically estimated length and volume of the clot correlated significantly with the clinician’s choice of more invasive therapies, i.e., the greater the clot length (*p* = 0.002, r = 0.42) and the higher the calculated volume (*p* = 0.033, r = 0.28), the more likely the clinician decided in favor of an invasive interventional thrombectomy rather than an exclusively supportive therapy.

## 4. Discussion

Our findings suggest that automated clot detection in NECT images yields higher sensitivity than the visual assessment of HAS, and is complementarily beneficial to the ASPECTS. Stroke imaging is of paramount importance and therefore it should be made more confident. In agreement with other studies, the low inter-observer agreement of HAS between the neuroradiologists in our study is not surprising, as the challenge of detecting clots in NECT scans is not trivial [29,30]. For this reason, an independent automatic tool that compensates for this could improve image analysis. Fortunately, many approaches (CTA, CTP, magnetic resonance imaging (MRI) with diffusions-weighted imaging (DWI) and gradient-echo sequences (GRE)) to this diagnosis exist. Corresponding to the HAS sign on CT, the susceptibility vessel sign (SVS) on susceptibility-weighted imaging (SWI) presents acute or subacute thrombi on MRI due to locally increased deoxyhemoglobin [31,32]. The detection rates of vessel occlusions were similar between SWI, time-of-flight MR angiography (MRA), gadolinium-enhanced MRA, and digital subtraction angiography, although SWI is superior in identifying the distal end of the thrombus and estimating thrombus length [33]. However, due to its higher availability and the required speed for pre-treatment diagnostics, CT imaging remains the leader in this field. Hence, different post-processing tools attempt to target vessel occlusion in major vessels [20,21,22,34,35,36]. The authors of these studies used volumetric image analysis considering the volume, length, clot burden score, vessel morphology, or degree of curvilinearity. Most of the published studies are based on CTA [36,37,38,39]. Only a few studies are based on NECT images, which constitute the only available imaging option in many smaller hospitals [20,21,22,35]. Al Kasab et al. investigated an automated clot detection algorithm based on NECT images with true-positive rates similar to ours but did not compare them to clot length/volume and therapy chosen and nor to the detection rate of HAS by human readers [35]. The other cited studies are more focused on the technical part of the clot detection algorithm and do not compare it to clinical data, outcomes, or therapeutic approaches [20,21,22].

The major limitation of HAS recognition consists of its variable attenuation. The latter depends on the clot composition. Earlier post-mortem studies demonstrated that thromboembolic stroke could be caused by white, red, and mixed-blood cell clots [40]. They presume that red clots and some of the mixed clots are more readily detectable than the white ones and thus an ipsilateral to contralateral HU ratio of <1.382 is optimal for detecting intraarterial thromboembolism [41,42]. One possible hypothesis is that the higher detection rate of blood clots by the tool compared to human readers is due to the underlying learning neural network. The input for our neural network is always a CT slice with additional information per voxel such as the HU difference to the opposite side. The advantage of this is that the algorithm does not necessarily rely on high-density values within the clots. In comparison to clot characterization in MRI, our results are in line with the literature. Liebeskind et al. and Kimura et al. demonstrated that erythrocyte-rich thrombi are associated with increased CT density and with increased susceptibility on T2*-weighted gradient echo images [6,17]. Furthermore, the susceptibility can be quantified using quantitative susceptibility mapping and higher susceptibility values which indicate a cardioembolic etiology with a higher diagnostic performance than SVS [43].

In the last years, neural networks were trained to learn thresholds for multilevel thresholding and optimized vessel segmentation [44,45]. In a recent report by Chung et al., the machine learning technique increased inter-rater reliability in interpreting stroke image data [46]. In their study, machine learning applied in GRE MRI scans using radiomics clot analysis provided valuable information on clot composition in acute MCA occlusion patients. In a similar attempt to ours, Qiu et al. focused on thrombus characterization in stroke patients using radiomics analysis performed in both NECT image data and CTA scans [47]. They could demonstrate that radiomics features derived from these image data sets were more predictive for recanalization with intravenous alteplase compared to more classical parameters like clot length, volume, or permeability.

The major strengths of our prototype are the higher sensitivity and PPV compared to the two readers. Like the readers, much more clots were detected in the anterior circulation compared to the posterior circulation (74% vs. 35%). Hence, clot detection for ICA, MCA1, and MCA2 was significantly superior to the detection rate by the readers. The reversal for MCA2 clots is plausible, as with increasing distance to the circle of Willis, the arterial caliber rapidly decreases, limiting clot depiction. A further advantage of the prototype software is the relative independence of clot detection from the scan plane. For human readers, HAS in arteries that run perpendicular to axial planes might avoid detection [48]. To address this issue, Mannel et al. propose to include multiplanar reconstructions and sagittal planes to increase the sensitivity of detection of the MCA “dot” sign [49]. Clot detection by the readers improved in patients with larger infarcts (lower ASPECTS), presumably due to better clot-to-background contrast. However, the automated clot detection of our prototype was not associated with the ASPECTS. Though we did not have a standard of reference for assessing the clot length and volume, the calculated values significantly correlated with the treatment invasiveness; thus, indirectly confirming the severity of the vessel occlusion as scored by the prototype.

To our knowledge, automated clot detection in acute ischemic stroke based on NECT image data is rare, and was not compared with clot characteristics and therapeutic decision-making to date. Its improvement for the smaller intracranial vessels should be pursued to make stroke diagnosis more reliable and quicker. Interestingly, the automatically estimated length and volume of the clot correlated with the clinician’s choice of more invasive therapies.

Our study has some limitations. First, the small number of patients only allows for a preliminary evaluation of this prototype. Further studies are needed to confirm the findings. Second, for the validation of the tool’s output for clot length and volume, we had to rely on an indirect correlation with the invasiveness of the applied treatment, as the CTA only shows the vessel occlusion site but cannot capture the entire clot length. Third, the prototype is technically unable to detect patients without vascular occlusions because the clot tool always suggests one to five clot candidates; thus, further studies are required to evaluate the tool’s ability to ensure correct negative classification and avoid false positives. Fourth, we investigated patients with a singular intracranial vessel occlusion. In contrast, every tenth patient with an acute ischemic stroke presented a multivessel occlusion which is associated with decreased endovascular success and a worse outcome [50]. Due to this relatively high prevalence, this multivessel occlusion scenario should be considered and needs to be investigated in further studies regarding the robustness and performance of the tool. Fifth, this prototype is not fully automated because the true clot must be manually selected by a (neuro)radiologist from a choice of five candidates weighted by likelihood.

## 5. Conclusions

Our preliminary results showed that the presented clot detection tool based on a convolutional neural network detected 56 of 85 intracranial clots, although it is technically unable to identify patients without occlusion. In particular, in ICA and proximal MCA, the tool demonstrated higher sensitivity and PPV than human readers. This new method can alert the treating neurologists and reporting radiologists of potential intracranial vessel occlusion, prioritizing the assessment of patient imaging and management. It could be especially beneficial to small hospitals or countries where CTA is not readily available or highly specialized neuroradiologists are limited.

## Figures and Tables

**Table 1:** Accuracy of clot detection. CTA = computed tomography angiography; ICA = intracranial internal carotid artery; MCA1/2/3 = proximal/middle/distal-middle cerebral artery; BA = basilar artery; PCA1/2 = proximal/distal-posterior cerebral artery.

Parameter	ICA	MCA1	MCA2	MCA3	BA	PCA1	PCA2	Total
Ground truth (CTA)	12	16	32	8	6	7	4	85 [100%]
True positive								
Tool	11	14	22	3	4	1	1	56 [66%]
Reader 1	5	7	14	5	5	3	0	39 [46%]
Reader 2	5	10	3	0	2	0	0	20 [24%]
False positive								
Tool	1	2	10	5	2	6	3	29 [34%]
Reader 1	1	1	6	2	0	1	1	12 [14%]
Reader 2	1	0	0	0	0	0	0	1 [1%]
Sensitivity in %								
Tool	92	88	69	38	67	14	25	66
Reader 1	42	44	44	63	83	43	0	46
Reader 2	42	63	9	0	33	0	0	24
Positive predictive value in %								
Tool	92	88	69	38	67	14	25	66
Reader 1	83	88	70	71	100	75	0	76
Reader 2	83	100	100	n/a	100	n/a	n/a	95

**Table 2:** Results of automated clot detection by the software, clot candidate score, and ASPECTS. ASPECTS = Alberta Stroke Program Early CT Score; CCS = clot candidate score; ICA = intracranial internal carotid artery; MCA1/2/3 = proximal/middle/distal-middle cerebral artery; BA = basilar artery; PCA1/2 = proximal/distal-posterior cerebral artery.

Location	ASPECT Score	Volume [mm^3^]	Longest Extent [mm]	CCS [Mean ± Standard Deviation]
	Median (Range)	Mean (Range)	Mean (Range)	All Positions
ICA	8 (2–10)	159 (33–443)	19 (9–37)	65 ± 20
MCA1	1 (0–9)	159 (1–365)	17 (1–41)	58 ± 23
MCA2	9 (2–10)	74 (2–332)	12 (2–36)	40 ± 24
MCA3	9 (0–10)	7 (6–8)	4 (3–5)	19 ± 13
BA	9 (7–10)	68 (13–137)	10 (4–21)	27 ± 22
PCA1	10 (7–10)	9 (9–9)	3 (3–3)	38 ± 54
PCA2	8 (4–9)	20 (20–20)	6 (6–6)	13 ± 0
Total	8 (0–10)	106 (1–443)	14 (1–41)	46 ± 27

## Data Availability

Not applicable.

## Appendix A

**Table A1:** Clinical data and location of vessel occlusion. CTA = computed tomography angiography; ICA = intracranial internal carotid artery; MCA1/2/3 = proximal/middle/distal middle cerebral artery; BA = basilar artery; PCA1/2 = proximal/distal posterior cerebral artery; NIHSS = National Institutes of Health Stroke Scale; TOAST = Trial of Org 10172 in Acute Stroke Treatment.

Parameter		ICA	MCA1	MCA2	MCA3	BA	PCA1	PCA2	Total
Total number based on CTA		12	16	32	8	6	7	2	85 [100%]
Side [r/l]	basilar	0	0	0	0	6	0	0	6 [7%]
	left	7	8	14	7	0	2	1	40 [47%]
	right	5	8	18	1	0	5	1	39 [46%]
NIHSS: median (range)		12 (0–23)	11 (1–22)	7 (2–21)	5 (1–21)	0 (32–11)	9 (4–22)	5 (2–19)	
Time interval between symptom onset and CT scan	<4.5 h	8	12	24	6	3	4	0	58 [68%]
	>4.5 h	4	1	2	0	1	3	1	12 [14%]
	uncertain	0	3	6	2	2	0	1	15 [18%]
Etiology of Stroke classified to TOAST criteria	large-artery atherosclerosis	5	2	6	0	4	0	1	19 [22%]
	cardioembolic	5	7	20	3	1	4	1	41 [48%]
	other determined etiology	1	1	2	2	0	0	0	6 [7%]
	undetermined etiology	1	6	4	3	1	3	0	19 [22%]
Atherosclerosis Score published by Chen et al. [24]	none	1	3	6	1	0	0	0	12 [14%]
	mild	3	10	5	2	4	1	0	26 [31%]
	mild to moderate	2	3	14	5	0	2	0	26 [31%]
	moderate	3	0	3	0	1	1	2	10 [12%]
	severe	3	0	4	0	1	3	0	11 [13%]
Therapy chosen	exclusively supportive	3	0	7	4	3	3	1	22 [26%]
	intravenous thrombolysis	4	1	14	4	1	3	0	28 [33%]
	interventional thrombectomy	4	5	1	0	1	0	0	11 [13%]
	thrombolysis and thrombectomy	1	10	10	0	1	1	1	24 [28%]

**Table A2:** Clot candidate distribution with corresponding Clot Candidate Score of automated clot detection by the software. ICA = intracranial internal carotid artery; MCA1/2/3 = proximal/middle/distal middle cerebral artery; BA = basilar artery; PCA1/2 = proximal/distal posterior cerebral artery.

Location	Clot Candidate Score [Number (Mean ± Standard Deviation)]									
	Position 1		Position 2		Position 3		Position 4		Position 5	
ICA	7	(77 ± 7)	3	(53 ± 17)	0	(0 ± 0)	1	(23 ± 0)	0	(0 ± 0)
MCA1	11	(67 ± 10)	1	(49 ± 0)	0	(0 ± 0)	0	(0 ± 0)	2	(9 ± 8)
MCA2	6	(68 ± 18)	9	(32 ± 16)	5	(28 ± 16)	2	(22 ± 15)	0	(0 ± 0)
MCA3	1	(33 ± 0)	0	(0 ± 0)	0	(0 ± 0)	1	(8 ± 0)	1	(15 ± 0)
BA	2	(49 ± 4)	0	(0 ± 0)	1	(29 ± 0)	0	(0 ± 0)	1	(9 ± 0)
PCA1	1	(76 ± 0)	0	(0 ± 0)	0	(0 ± 0)	0	(0 ± 0)	0	(0 ± 0)
PCA2	0	(0 ± 0)	0	(0 ± 0)	1	(13 ± 0)	0	(0 ± 0)	0	(0 ± 0)
Total	28	(68 ± 14)	13	(38 ± 18)	7	(26 ± 15)	4	(19 ± 11)	4	(10 ± 6)
